# Properties Investigation of GO/HA/Pt Composite Thin Film

**DOI:** 10.1155/2019/4847932

**Published:** 2019-06-20

**Authors:** Huda F. S. G. Alyafei, W. Fu, E. Zalnezhad, F. Jaber, A. M. S. Hamouda, F. Musharavati, S. Bae

**Affiliations:** ^1^Mechanical and Industrial Engineering Department, College of Engineering, Qatar University, 2713 Doha, Qatar; ^2^Department of Mechanical Engineering, Hanyang University, 222 Wangsimni-ro, Seongdong-gu, Seoul 133-791, Republic of Korea; ^3^Biomechacin LLC, San Antonio, TX 78251, USA; ^4^Department of Biomedical Engineering, Ajman University, UAE; ^5^Department of Architectural Engineering, Hanyang University, Seoul 04763, Republic of Korea

## Abstract

Hydroxyapatite/graphene oxide/platinum (HA/GO/Pt) nanocomposite was synthesized and electrodeposited on a pure zirconium substrate. The coated zirconium was annealed at 200, 300, 400, and 600°C in vacuum furnace in presence of argon gas. The structure and morphology of the coated samples were characterized. Biocompatibility and wear and corrosion resistances of specimens were examined. The result of corrosion tests shows that the graphene into HA/Pt composites significantly improves their corrosion resistance. The wear tests results of uncoated and coated samples before and after annealing show that coated samples annealed at 300°C had better wear resistance compared with uncoated and coated samples at other temperatures. Furthermore, the biocompatibility test shows that the coatings improved the cell attachment and proliferation compared to the pure zirconium substrate.

## 1. Introduction

Nowadays, the medical practice uses a large number of implants and devices. Medical devices (blood tubes, artificial hearts, biosensors, and pacemakers) and implants (bone plates, ligaments, joint replacements, heart valves, intraocular lenses, vascular grafts, dental implants, and sutures) are made of biomaterials extensively utilized to restore and/or replace the function of degenerated or traumatized organs or tissues, to correct abnormalities, to improve function, to help in healing, and consequently to improve the patients' quality of life [1].

Today, one of the most challenging tasks for materials science is the development of new biomaterials for medical applications. The necessity of better implants is an obvious need and thus the manufacturing of artificial tissues is highly demanded [2].

Being biocompatible with no toxicity or harm to tissues in the human body is one of the most vital factors that differentiates biomedical materials from other materials. For many years, the development and sustainability of mutually acceptable coexistence of tissues and biomaterials have been of interest to scientists in the field of biomaterials and medical devices users [3].

Concentrations of interstitial fluid and chloride ions in serum are an extremely corrosive environment for biometallic materials. Body fluid (so-called electrolytes) comprised numerous proteins and amino acids that corrode metallic materials. Furthermore, the dissolved oxygen concentration in body fluid is small, which leads to delay the regeneration of the oxide layer on metallic biomaterials, and when the film is detached a large number of metal ions are released [4, 5].

Hence, to improve the performance of implants in vitro and in vivo, it is essential to control the interfacial behaviors between the implant and host. Surface treatment can successfully change the chemical and physical properties of the surface of biomaterials to develop the functionalities and properties of the original materials and therefore decrease the opposing interfacial interactions between implants and their surrounding biological environment. For decades, frequent surface treatment techniques have been established to eventually upsurge the hemocompatibility and biocompatibility of zirconium based alloys, comprising the deposition of polymer coatings or inorganic thin films (zirconium nitride, diamond-like carbon, zirconium oxide, etc.) and the fabrication of a multifunctional layer for the immobilization of biomolecules using the wet chemical method [6–9]. Considerable progress has been attained in blood compatibility and biocompatibility.

So far, numerous works have been performed to develop bioactivity and corrosion resistance of Zr and its alloys for biomedical applications utilizing a number of surface modification techniques such as the sol–gel process, chemical or physical vapor deposition, anodization, thermal oxidation, and plasma electrolytic oxidation [10, 11].

Hydroxyapatite (HA) with the formula Ca_5_(PO_4_)_3_(OH) is the main mineral composition in human bone and teeth [12] and it is, therefore, a useful bioceramic. The favorable biocompatible properties of hydroxyapatite are well documented. It bonds to bone forming indistinguishable unions and it is rapidly integrated into the human body [13].

A great number of papers are available for dealing with different systems based on graphene oxide, hydroxyapatite, and other composites with the aim to be applicable as biomaterials [8, 14–16].

In this study, nanocomposite coating based on HA, reduced graphene oxide, and pure platinum were deposited on zirconium substrates using the electrodeposition technique. The goal was to gain a hybrid ceramic-metal coating, reinforced with graphene oxide that will simultaneously ensure an increase in biocompatibility and corrosion and wear resistances. Surface characterizations were carried out to evaluate the morphology and structure of the coatings using XRD, TEM, SEM, and FTIR.

## 2. Experimental Procedure

Commercially available zirconium substrates of 99.5% purity (Alfa Aesar, Heysham, England) with dimensions of 40 × 10 × 0.127 mm^3^ were used as substrates for electrodeposition. The surface of the zirconium substrate was ground and polished with silicon carbide sandpapers (grit range of 800-1500) and etched in an HF solution for 1 h. Then, it was ultrasonically (Daihan Ultrasonic Bath Set WUC-A02H) cleaned in ethyl alcohol, acetone, and deionized water for 30 min and lastly dried in a vacuum oven at 70°C.

Hummers method was used to prepare Graphene oxide [17]. Baradaran et al. used Hummers method for preparation of rGO to investigate the biocompatibility of a nanotube hydroxyapatite-reduced graphene oxide composite [18]. In our work, once GO's synthesis process completed, the suspension was rinsed with dilute 1 M HCl and deionized water several times to reach a pH of 5. The product was separated from the mixture centrifugally at 11,000 rpm. Furthermore, to remove unwanted materials from the final product which could affect the biocompatibility of the HA/GO/Pt composite, the GO was mixed with dd H_2_O and centrifuged five times at 4500 rpm for 10 min. The EDX results detected no trace of other bioincompatible elements used during the Hummers method.

To obtain a golden and transparent GO, 100 ml distilled water was added to 20 ml graphene oxide and mixed thoroughly followed by ultrasonic treatment in double distilled water for 2 h at 570 W.

To attain the hydroxyapatite solution, 2.45 g CaCl_2_ and 0.58 g NaCl were added into 50 ml of distilled water and mixed using a magnetic stirrer. Then, 1.150 g NaH_2_H_2_PO_4_ (Sigma-Aldrich 99.5% trace metals basis) and 50 ml of distilled water were separately mixed by a magnetic stirrer. Finally, the two solutions were mixed and stirred to obtain a hydroxyapatite solution.

To prepare 0.001 mol/L Pt solution, 0.01g K_2_PtCl_4_ powder was added to 25 ml distilled water in an opaque flask.

**Figure 1:**
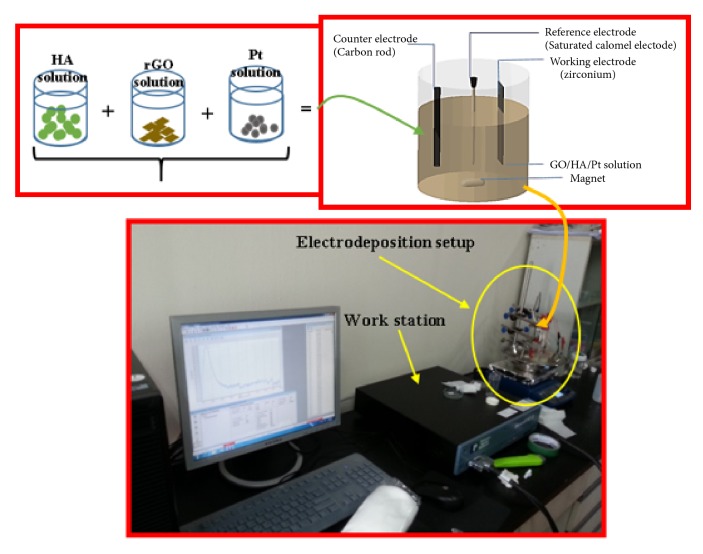
Experimental setup for electrodeposition.

Electrodeposition method was used to deposit the composite layer onto the substrate. 40 ml hydroxyapatite, 40 ml graphene oxide, and 25 ml Pt solutions were mixed and stirred for 30 min. Then, the pH of the resulting solution was adjusted to 6 using 1 M NaOH in a water-bath at 80°C. The electrodeposition (Potentiostat/Galvanostat/Impedance Analyzer, PARSTAT300) was performed in a standard three-electrode cell using a chronoamperometry cycle in which pure zirconium substrate, a graphite rod, and a saturated calomel electrode (SCE) were the working electrode, the counter electrode, and the reference electrode, respectively (see Figure 1). The electrolyte was stirred smoothly at 80 rpm during electrodeposition. The applied potential was kept at −2 V for 7200 s (time per point was 2 s with total points of 3600). Finally, the electrodeposited samples were washed with deionized water and dried in an oven at 70°C.

The electrochemical behavior of the uncoated, HA-, GO-, HA/GO-, and HA/GO/Pt-coated samples were studied in a CaCl_2_ solution. A conventional three-electrode electrochemical cell with a platinum wire as a counter-electrode, a saturated calomel as a reference electrode, and the specimen, which served as the working electrode, were used. To let the open circuit potential (OCP) become stable, the samples were kept in the solution for 2 h before the electrochemical corrosion test. Potentiodynamic polarization tests were carried out, starting at −250 mV with reference to the OCP at a sweep rate of 0.5 mV−1 to a final current density of 0.1 mAcm^−2^. Electrochemical impedance spectroscopy (EIS) was conducted at the OCP with an AC amplitude of 10 mV over the frequency range of 10,000–0.01 Hz.

For the biocompatibility tests, to visualize the viable cells, the cell line MDA-MB-231, comprising a constitutively active GFP expression system, was used. Dulbecco's modified Eagle's medium (DMEM) with 1% pen strep (PS) antibiotic and 10% fetal bovine serum (FBS) was used for cell culture process in T-25 culture flasks using. Detailed info can be found in the study performed by Zalnezhad et al. [19].

**Figure 2:**
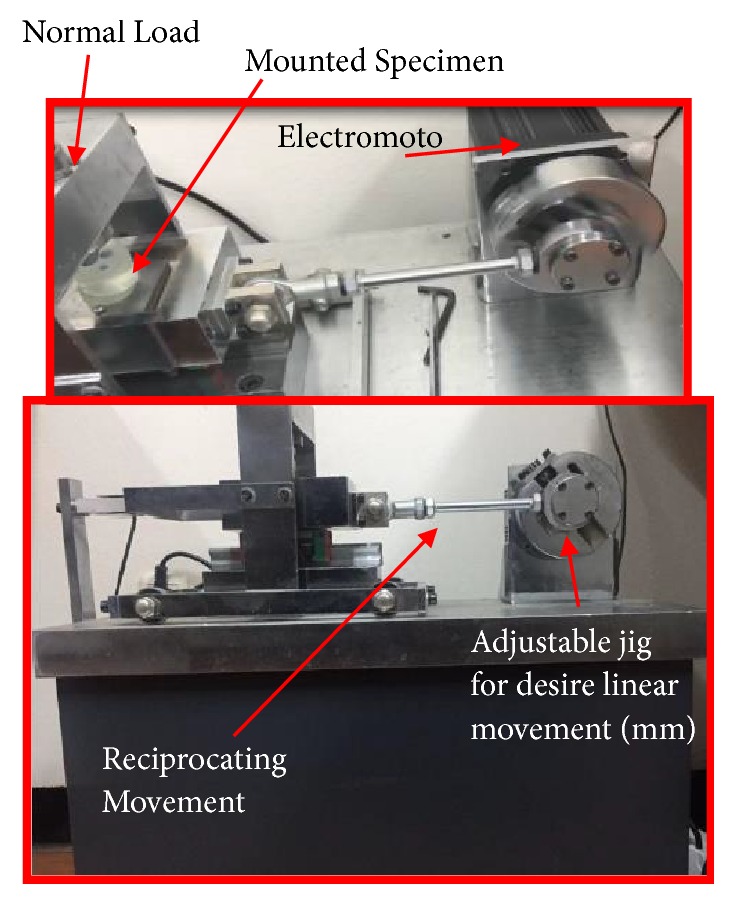
Reciprocating wear test setup.

The wear resistance tests of the coated samples were performed using a reciprocating ball-on-disc wear test machine. A 6 mm stainless steel ball was used for wear test at ambient temperature. In this experiment, the sliding speed and the track diameter were 1 cm/s and 5 mm, respectively. A 4 N normal load was applied in this work (see Figure 2). Five experiments were carried out for each sample and friction coefficients of thin film deposited sample were recorded.

Furthermore, the morphology and structure of the deposited layer were characterized using FESEM (FEI, USA). FT-IR tests were performed utilizing Nicolet iS50 FT-IR spectrometer in which the spectral resolution and the scan range were 0.5 cm^−1^ and 450-4000 cm^−1^, respectively. XRD (Smart Lab, Rigaku) peak intensity was measured with the scan-step of 2*θ* = 20–80, and a 0.02 step width in which the exposure time was 50 s per step. TEM was utilized to study the elemental composition and the morphology of the coatings (JEM-2010 TEM (JEOL, Japan).

**Figure 3:**
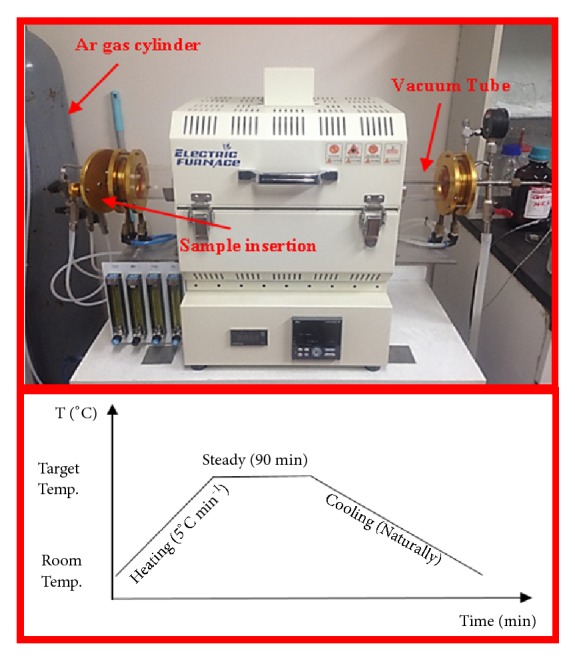
Tube furnace along with the heating and cooling graph.

To evaluate the influence of heat treatment on morphology and properties of coated substrates, a tube furnace equipped with Ar gas was used for the heating process of HA/GO/Pt coated samples at different temperatures including 200, 300, 400, and 600°C. The vacuum pressure and the Ar gas flow rate were 1.0 × 10^−3^ torr and 200 sccm, respectively. The heating rate of 5°C min^−1^ was selected until the desired temperature was achieved for 1.5 hr. Finally, the heated specimens were left at room temperature to cool naturally (see Figure 3).

## 3. Results and Discussion

### 3.1. Reaction Mechanism of Electrodeposition

**Figure 4:**
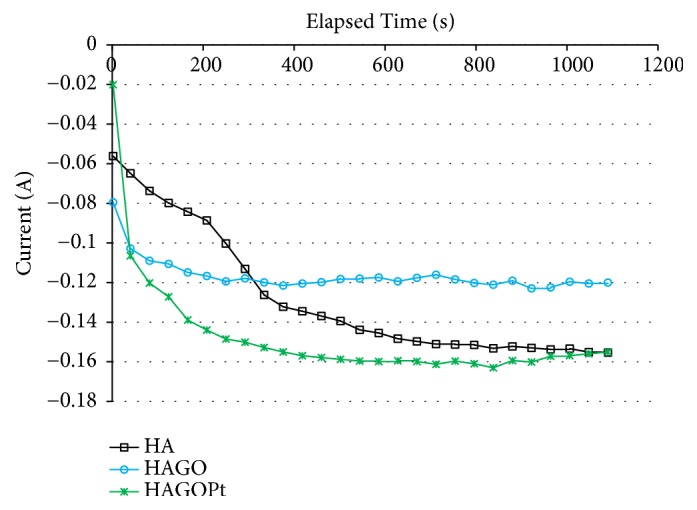
Chronoamperometry plot of electrodeposition process of different coatings.

Figure 4 shows the variation of the current with time during the coating process. It can be seen from the figure that the electrolyte within the active material began to react electrochemically within 2 minutes. The second minute to the fourth minute the current density is slightly reduced, which indicates that the nucleation mechanism of the particles on the zirconium surface, this process is controlled by electron transfer. Note that the current density was reduced from 4 minutes to 10 minutes, which means that the deposited particles are coated on the zirconium surface by mass transfer and electron transfer processes. After 10 minutes to 30 minutes, due to the well transportation process, the current density tends to be stable. After 30 minutes, a large number of bubbles were observed on the zirconium surface, which is due to the hydrogen ions in the solution. Hydrogen spills in the electrodeposition synthesis which would affect the adhesion of the coating and the substrate and it would cause the coating to be uneven. Therefore, in order to synthesize an excellent coating which is not disturbed by hydrogen on the zirconium surface, the deposition time should be selected as 10 to 30 minutes. The electrodeposition time was 18 minutes in our investigation.

### 3.2. Structural and Morphological Analysis

**Figure 5:**
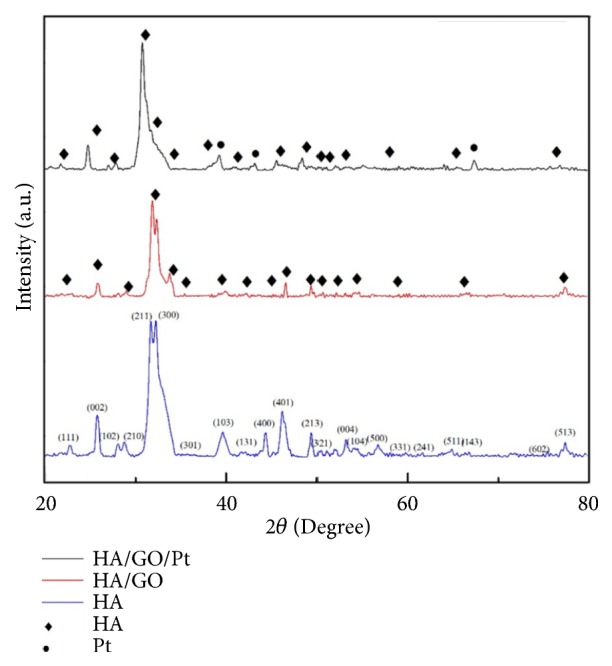
X-ray diffraction patterns for the synthesized HA, HA/GO, and HA/GO/Pt.

XRD patterns for HA, HA/GO, and HA/GO/Pt composite coatings are presented in Figure 5. The XRD diffraction peaks of the three samples can be found at (002), (211), (103), (401), and (213) that represents the typical structure of hydroxyapatite (JCPDS 09-0432). The diffraction peaks of (111), (220), and (200) appearing in the HA/GO/Pt sample indicate that the HA/GO/Pt composite was successfully synthesized (JCPDS 04-0802). In addition, the diffraction peaks of HA decreased after adding the GO and GO/Pt. The three most intensive peaks of (300), (103), and (401) between 30° and 45° (2*θ*) in a standard diffraction curve are not obvious which are possibly caused by low crystallinity or nanometer scale distribution. It is clear that the crystal size of hydroxyapatite is small from the sharp diffraction peaks [20, 21].

**Figure 6:**
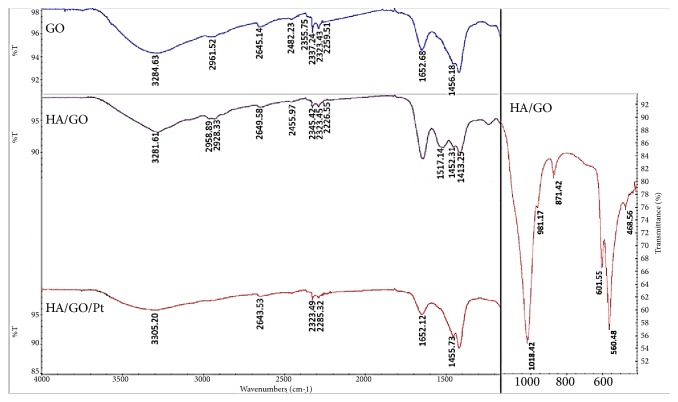
FT-IR spectra of GO, HA/GO and HA/GO/Pt coatings on zirconium.

The characteristic absorption bands and corresponding wave numbers of GO, HA/GO, and HA/GO/Pt coatings are indicated using FT-IR shown in Figure 6. The mutual absorbance bands at approximately 3285 cm^−1^ are assigned to the hydroxyl group (OH^−^) stretching. The position of the characteristic bands at 1018.42, 981.17, and 560.48 cm^−1^ in the FT-IR is attributed to the stretching and bending of phosphate [22]. The characteristic band at 601.55 cm^−1^ is attributed to the vibrational mode of the OH^−^ group in the HA structure [23, 24]. The band at 871.42 cm^−1^ is assigned to the P-(OH) stretching vibration in the HPO_4_ 2- phosphate group [25, 26]. The bands at 1653 cm^−1^ and 1456 cm^−1^ are assigned to the stretching vibrations of the carboxyl group (COOH-) on the edge of the basal planes or the conjugated carbonyl groups (C=O) and the sp2 hybridized C=C vibration stretching, respectively [27]. The absorption bands of the methylene groups (CH_2_), which are inherent in the GO, are present at approximately 2959 cm^−1^ and 2928 cm^−1^. The peak at 1425 cm^−1^ is attributed to the deformation of the O–H [28]. In contrast, the peaks at 1750 cm^−1^ and 1425 m^−1^ in the FT-IR spectrum of the HA/GO composite are no longer visible, which points to the reduction of GO.

**Figure 7:**
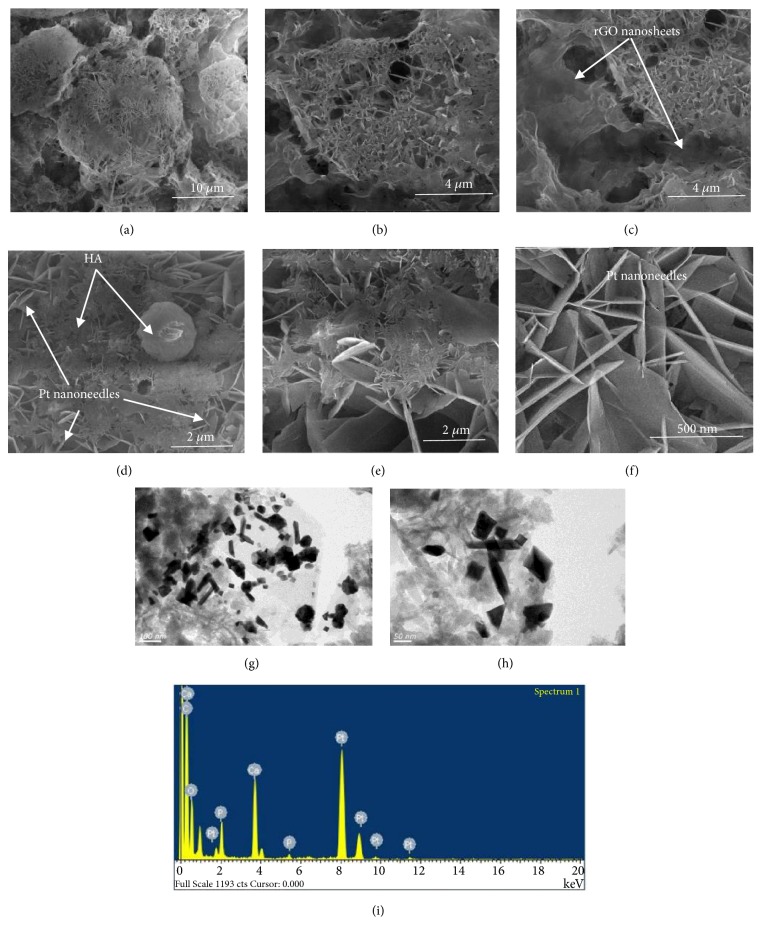
FESEM images-top views (a-f), TEM (g and h), and EDS (i) of HA/GO/Pt.

Structural analysis and surface morphology of electrodeposited thin film HA/GO/Pt nanocomposite over zirconium substrate were performed using SEM and TEM. Figures 7(a)–7(f) show SEM micrographs of HA/GO/Pt coating at different magnifications. The surface of the pure zirconium substrate is covered by hydroxyapatite particles, Pt nanowalls, and graphene nanosheets all over the substrate. As can be seen, the coating is porous. An SEM micrograph of the composite coating (Figure 7(a)) shows the characteristic morphology for HA agglomerates with different sizes. The SEM micrograph of the HA/GO/Pt composite coating (Figure 7(c)) shows HA particles and nanowalls wrapped by reduced graphene oxide nanosheets. Incorporation of graphene changes the morphology of the composite coating significantly.  Figure 7(e) shows wave-like graphene sheets, well dispersed in a broccoli-looking HA/Pt. In addition, in HA/GO/Pt a distribution of HA particles in the Pt/graphene matrix is observed. HA and graphene are connected by Van der Waals bonding [29]. Therefore, HA crystals nucleation is perhaps created on either the cross-section of graphene multi-sheets or the graphene wall, followed by succeeding crystal growth perpendicular or along the graphene sheet's surface.  Figure 7(g) and h show the TEM at different magnifications and Figure 7(i) shows the EDS elemental analysis of HA/GO/Pt composite. As can be seen the composite coating consists of Ca, P, Pt, O_2_, and C elements in EDX indicating the presence and contribution of Pt (~33.38 wt.%), HA nanowalls (~32.96 wt.%), and graphene oxide nanosheets (~20.28 wt.%) in the HA/GO/Pt composite thin film.

**Figure 8:**
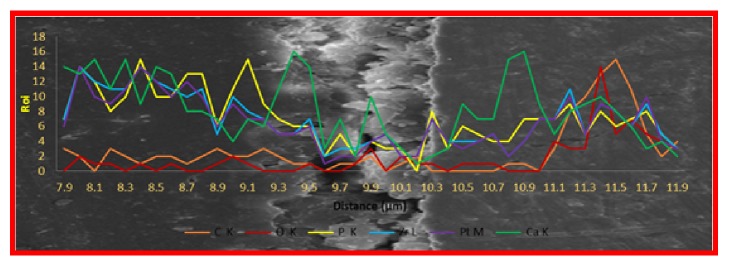
Cross-sectional FESEM micrographs and elemental profile plot of HA/GO/Pt coating.

Figure 8 shows a cross-sectional FESEM-EDX micrograph of an HA/GO/Pt coating on the zirconium substrate in which the thickness of the composite thin layer is found to be around 1.1 *μ*m. The deposited layer was uniform in structure and there were no 3D microscopic bulk defects. In the EDS analysis, line scanning method from the cross-section was carried out to detect the elemental composition the coating to confirm the creation of HA/GO/Pt thin film on the zirconium substrate. From the EDS line scanning, elements such as C, O, Pt, P, and Ca are detected over the substrate, indicating that they are incorporated into the surface structure during electrodeposition. It further confirmed the formation of HA/GO/Pt composite coatings on the zirconium substrates.

### 3.3. Biocompatibility Tests

**Figure 9:**
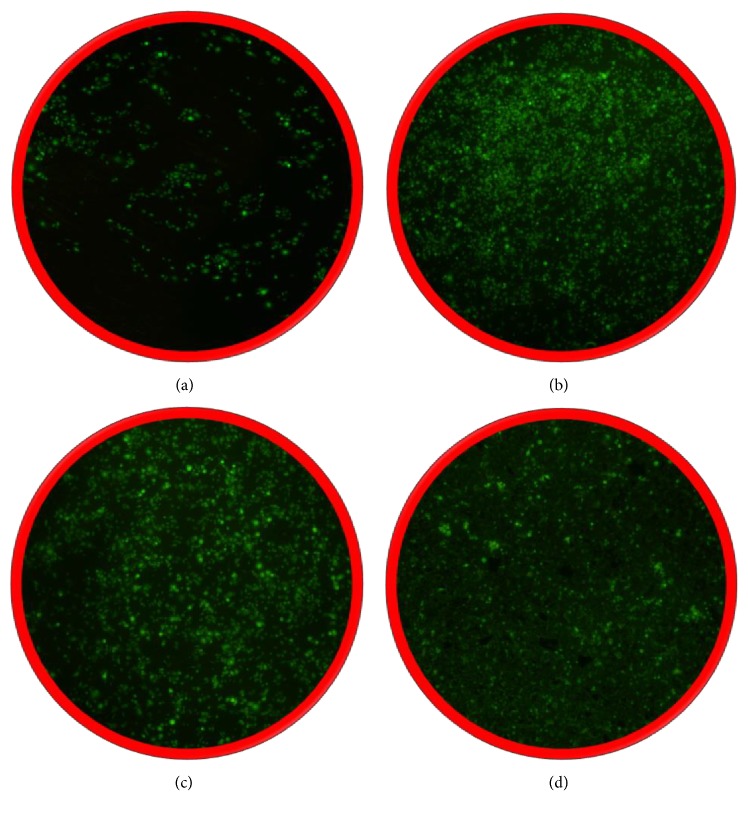
Biocompatibility of the (a) uncoated and (b) HA, (c) HA/GO, and (d) HA/GO/Pt coated substrate using the human cell line MDA-MB-231 possessing a green fluorescent protein as a reporter for living cells.

Figures 9(a)–9(d) present the biocompatibility tests of uncoated and HA-, HA/GO-, and HA/GO/Pt-coated zirconium. In looking at the biocompatibility, the uncoated zirconium surface was not capable of providing extensive attachment and growth of the adenocarcinoma cells. In stark distinction, the HA and HA/GO coating surfaces allowed cell attachment and proliferation as was observed by the bright fluorescent signal from the green fluorescent protein (GFP) produced within the viable MDA-MB-231 cells. In addition, the cell spreading on the surface was indicative of a characteristic epithelial morphology suggesting strong adhesion and biocompatibility of the surface. The biocompatibility of the HA/GO/Pt surface similarly permitted cell spreading and proliferation. From Figures 6(a)–6(d), it can be realized that the cell proliferation and distribution on the HA/GO/Pt coating decrease compared to HA and HA/GO coatings, but it is better than bare Zr. It can be concluded that Pt may have no a significant effect on biocompatibility and cell proliferation in HA/GO/Pt composite. In our previous study [30], we found that the bare stainless steel 304 surface had no significant cells growth and attachment. But, SST 304 surface deposited by HA allowed cell proliferation and attachment. But a better growth in biocompatibility was seen for HA/rGO thin layer. Furthermore, the HA/rGO/Pd deposited layer onto stainless steel 304 surface allowed even more enhanced cell proliferation and spreading, concluding that Pd may have a better and more significant influence on the biocompatibility of the composite compared to the Pt element.

### 3.4. Electrochemical Measurements in CaCl_2_ Solution

In order to evaluate the corrosion stability in physiological media, EIS and PDS measurements in a CaCl_2_ solution were performed.

#### 3.4.1. Electrochemical Impedance Spectroscopy

**Figure 10:**
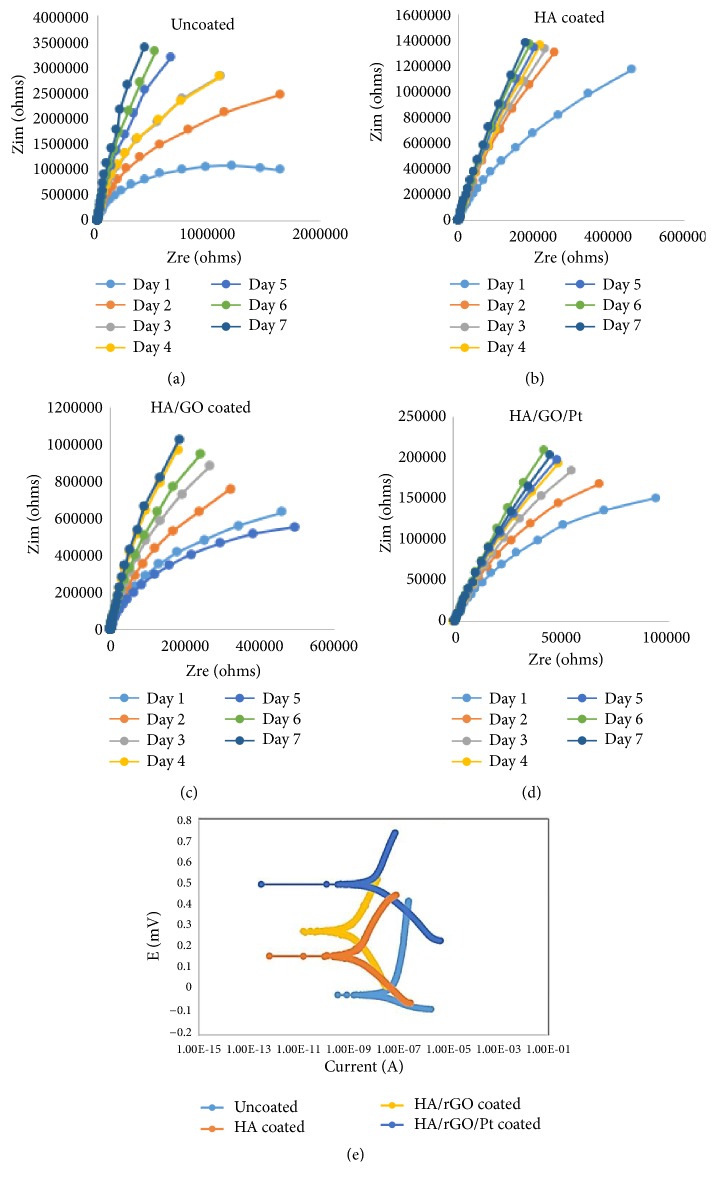
EIS spectra of (a) pure zirconium, (b) HA, (c) HA/GO, and (d) HA/GO/Pt coatings on zirconium substrate during prolonged time (7 days). (e) Potentiodynamic polarization curves of uncoated and coated zirconium after 7 in CaCl_2_ solution.

Nyquist plots of pure zirconium, zirconium coated with HA, HA/GO, and HA/GO/Pt coatings after different immersion times in CaCl_2_ solution are shown in Figures 10(a)–10(e), respectively. In general, the high-frequency range of the Nyquist plots is attributed to the coating, while the low frequency range describes electrochemical processes on the metal surface beneath the coating. The EEC contains the coating pore resistance R_c_, the electrolyte resistance R_s_, and constant phase elements CPE_ox_ and CPE_c_ that represent all of the frequency-dependent electrochemical phenomena, including the capacitance of the passive oxide layer on the Zr surface beneath the coating C_ox_ and the coating capacitance C_c_ [31].

Potentiodynamic sweep measurements in CaCl_2_ was performed to get information about the corrosive properties of the uncoated, HA-, HA/GO, and HA/GO/Pt-coated substrates, since the corrosion rate is proportional to the current density. Potentiodynamic polarization curves of the uncoated, HA-, HA/GO, and HA/GO/Pt-coated substrates after 7 days in CaCl_2_ solution are plotted in Figure 10. The corrosion current density I_corr_ and corrosion potential E_corr_ were evaluated according to Tafel extrapolation and are listed in Table 1. The E_corr_ of pure zirconium (-21.699 mV) and HA coating (164.574 mV) is less positive than the E_corr_ of HA/GO (267.02 mV) and HA/GO/Pt (499.325 mV). However, I_corr_ of bare pure zirconium, HA, HA/GO, and HA/GO/Pt is 279.602, 72.1, 50.044, and 27.227 nA, respectively. I_corr_ of HA/GO and HA/GO/Pt is lower than I_corr_ of HA and uncoated substrate, implying that graphene and Pt improve the corrosion resistance of the Zr substrate in CaCl_2_ solution as a consequence of the bioactivity of the formed apatite layer on HA/GO- and HA/GO/Pt-coated surface.

The results of polarization measurements are in accordance with the impedance spectroscopy results, indicating that the HA/GO and HA/GO/Pt coatings have better corrosion resistance and the lowest corrosion rate due to the thick biomimetic apatite layer on their surfaces. Therefore, the use of graphene-based HA/GO and HA/GO/Pt composite coatings may improve the corrosion resistance, decrease metal ion release. Fadol et al. evaluated the influence of HA/rGO/Pd on the corrosion behavior of SST304. They found that the uncoated SST 304 had E_corr_ = 0.13V, with a quite high current density, though the EIS test evidently showed that the electrodeposited SST 304 results in very small I_corr_ compared to the bare substrate. The lower I_corr_ was attributed to the contribution of graphene oxide nanosheets into the solutions that enhanced the rate of the electrodeposition and resulted in thicker film. Owing to the impermeable to molecules and chemical inertness of graphene, it plays a significant role as every corrosion resistance layer on a metallic material by performing like a natural diffusion obstacle. Inclusion of palladium in HA/rGO inhibited the corrosion process of the sample, probably because of the existence of palladium at grain boundaries that helps the corrosion resistance of the substrates [30]. A comparative investigation on the effect of HA/GO/Pd and HA/GO/Pt thin film layers shows that although, in the previous study, the HA/GO/Pd composite layer improved the corrosion resistance of SST304 significantly, HA/GO/Pt presents a better corrosion behavior for pure zirconium substrate. This may be due to the presence and better contribution of the Pt element as a barrier at grain boundaries in HA/GO/Pt composite layer.

#### 3.4.2. Corrosion Behavior and Surface Morphology of the Thermal Treated HA/GO/Pt Coated Zirconium

**Figure 11:**
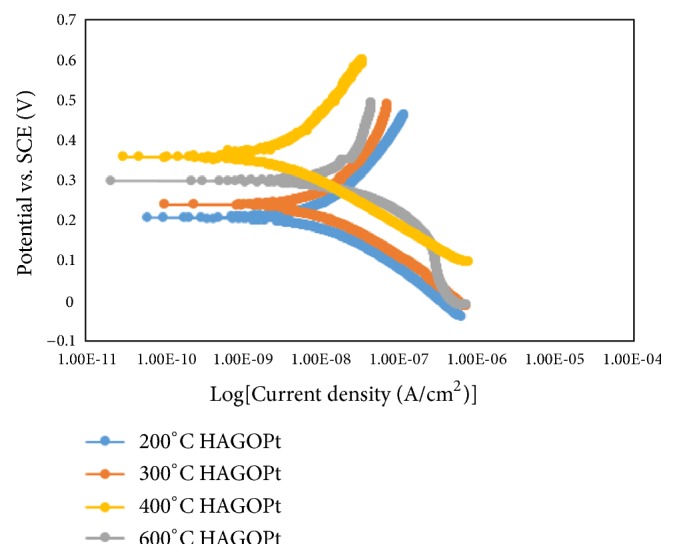
The corrosion behaviors of the thermal treated HA/GO/Pt coated zirconium.

Figure 11 shows the polarization curves of HA/GO/Pt nanocomposites heat treated at 200, 300, 400, and 600°C.  Table 2 summarizes their respective E_corr_ and I_corr_. The corrosion potential, E_corr_, of HA/GO/Pt coated samples annealed at 200, 300, 400, and 600°C is 1.731, 232.858, 379.096, and 309.423mV, respectively. The results show that HA/GO/Pt coated substrate annealed at different temperatures has E_corr_ lower than E_corr_ of the untreated HA/GO/Pt coating, indicating that the heat treatment has a significant effect on corrosion potential of the composite coating. The current densities of the HA/GO/Pt coatings heat treated at 200 and 300°C were 16.269 nA and 17.035 nA, which were also lower than the current density of the untreated HA/GO/Pt coatings (27.227 nA). This indicates that the corrosion resistance of HA/GO/Pt coatings is enhanced by heat treatment. The higher corrosion potential (E_corr_) and the lower corrosion current density (I_corr_) indicate that the material has better corrosion resistance. For the heat treatment temperature of 400°C (E_corr_ 379.096 mV I_corr_ 1.521 nA), it achieved the best corrosion resistance. As can be seen, with increasing the annealing temperature from 200 to 400°C I_corr_ decrease showing a better corrosion resistance and that can be caused by the diffusion of coatings into the substrate and integration of the composite materials during the heat treatment process. Further increasing in temperature to 600°C led to increasing in I_corr_ = 14.15 indicating a decrease in corrosion resistance which can be due to cracks in the coatings or coating detachment because of different modulus of elasticity between the coating and the substrate. In the previous study by Fadol et al. [28] HA/GO/Pd was heated at versatile temperatures (200-600°C) and 200°C heating of HA/rGO/Pd showed better corrosion potential and current density compared to the other heat treated HA/GO/Pd. Compared with the nonannealed and other annealed samples, the E_corr_ for 200°C annealed HA/rGO/Pd was more positive and the plot had a tendency toward lower current density. This severe change in electrochemical behavior of the composite electrodeposited substrate may be due to the penetration of electrolyte into the defects of the thin layers caused at higher temperatures. This indicates that annealing of HA/rGO/Pd electrodeposited substrate at lower temperature has a better protective effect. Furthermore, heat treatment of HA/GO/Pt at 400°C shows a better corrosion behavior for pure zirconium substrate compared to HA/rGO/Pd coated SST304 annealed at 200°C (showed the best corrosion resistance).

**Figure 12:**
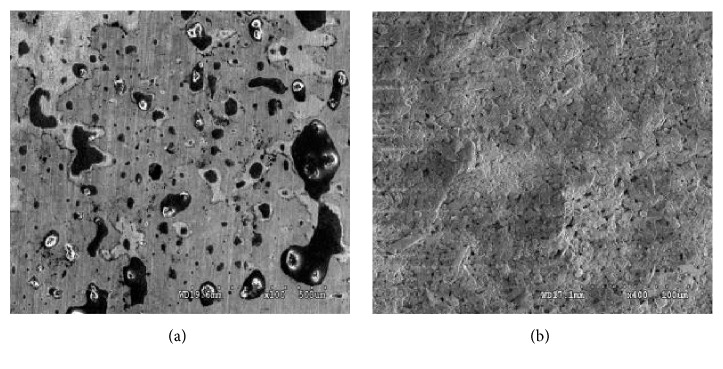
SEM micrographs of (a) bare and (b) HA/GO/Pt coated sample before and after corrosion test.

**Figure 13:**
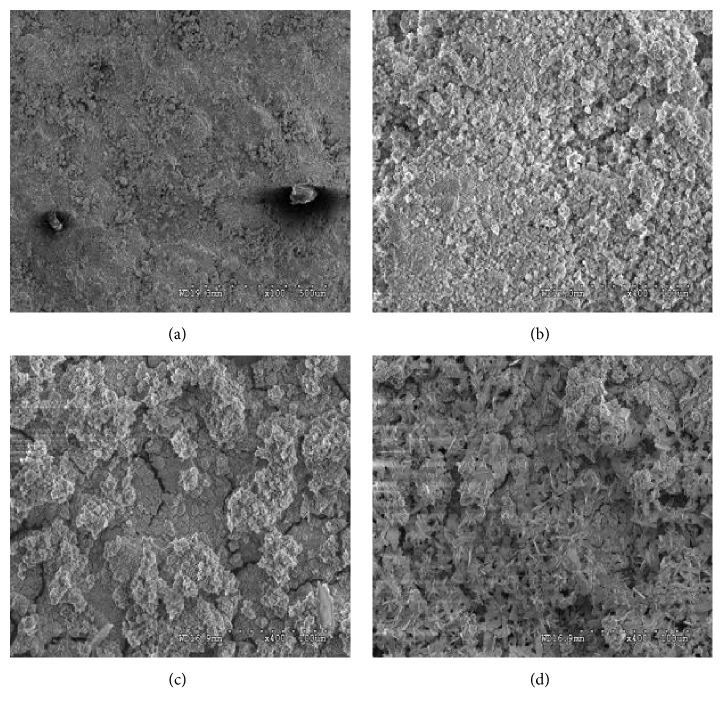
SEM micrographs of HA/GO/Pt coatings before and after corrosion tests (a, b) annealed at 200°C and (c, d) annealed at 600°C.

Figures 12(a) and 12(b) present the surface of the nonannealed bare and HA/GO/Pt coated zirconium substrates immersed into the CaCl_2_ solution for 7 days. As can be seen, the corrosion spots of the coated samples are less than bare samples one. It indicates that the coating can effectively improve the corrosion resistance. To better investigate the influence of the low (200°C) and high (600°C) temperatures on morphologies of the thin film coated substrate before and after EIS tests, the SEM analysis conducted and the results are depicted in Figures 13(a)–13(d). As can be seen from Figure 13(c), increasing in the annealing temperature to 600°C led to the creation of cracks all over the coatings due to the difference in elastic moduli in coating materials and the substrate resulting in a decrease of corrosion resistance as the corrosion solution penetrated to the substrate through the cracks. It can be concluded that high annealing temperature such as 400°C may have a better influence on corrosion resistance of HA/GO/Pt composite.

**Figure 14:**
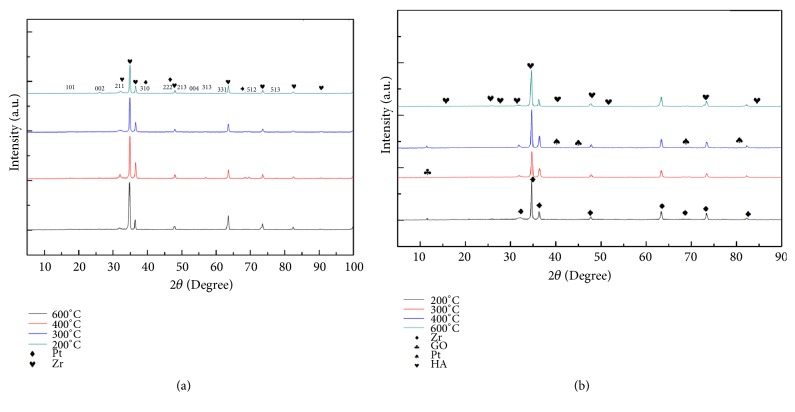
The phase analysis of the heat treated HA/GO/Pt coatings before (a) and after (b) corrosion test.

Figure 14 shows the XRD patterns of annealed HA/GO/Pt deposited zirconium substrate before and after EIS tests. Hydroxyapatite peaks with high intensity at (002) and (211) crystal planes at 25.8° and 32.0° show perfect match with the hydroxyapatite pattern (PDF#09-0432). Furthermore, the XRD analysis after soaking the coated samples in the corrosion medium for seven days shows the formation of the carbonated hydroxyapatite as the angle of crystal planes (002) and (211) shifted. The formation of the carbonated hydroxyapatite is favorable as it characterizes the mineral phase in human bone. The availability of the functional groups in the biomimetic synthetic serum can help the growth of the hydroxyapatite on the electrodeposited surface [32]. The hydroxyapatite surface charges negatively and thus attracts ions of Ca+2 by the exposure of the phosphate and hydroxyl ions in hydroxyapatite to the biomimetic synthetic serum. Calcium phosphate precipitates due to the high calcium ions consumption. Simultaneously, hydroxyapatite dissolves, enhancing the phosphate and calcium concentrations in the synthetic solution, which results in hydroxyapatite precipitation. The reaction during calcium phosphate dissolution and precipitation in the biomimetic synthetic medium is reversible [33]. Because of the negative surface charge of graphene coming from p electrons proliferation in sp2 hybrid orbitals, graphene can enhance the hydroxyapatite deposition rate by attracting ions of calcium [34].

### 3.5. Friction Coefficient and Wear Analysis

**Figure 15:**
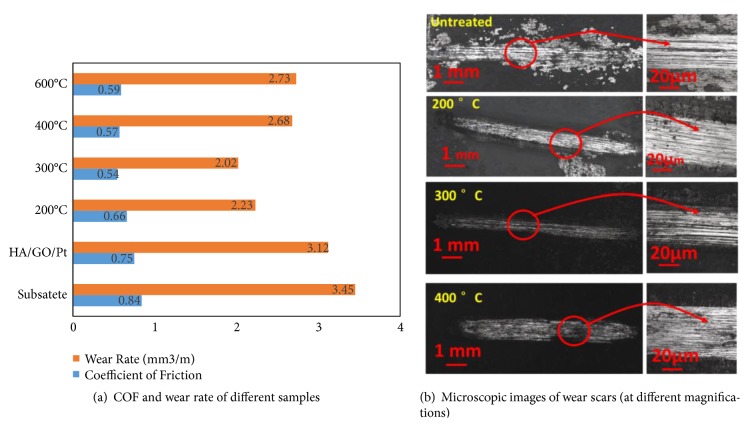
The friction coefficient and wear loss of substrate, HA/GO/Pt coating (before annealing) and HA/GO/Pt coatings after heat treatment at different temperatures.

Figure 15 presents the COF and wear loss of bare substrate, HA/GO/Pt (before annealing), and heated HA/GO/Pt electrodeposited zirconium at different heating temperatures. It is clearly evident that COF of HA/GO/Pt deposited zirconium affects by annealing at different temperatures. Average COF of the bare substrate, HA/GO/Pt (before annealing), and annealed HA/GO/Pt coated substrates at 200, 300, 400, and 600°C are 0.84, 0.75, 0.66, 0.54, 0.57, and 0.59, respectively. Definitely, the COF of electrodeposited zirconium heated at 300°C presents a significantly lower average COF compared to other specimens.

The wear rates of the pure substrate and HA/GO/Pt (before annealing) and heat treated HA/GO/Pt coated substrate at 200, 300, 400, and 600°C are 3.45, 3.12, 2.23, 2.02, 2.68, and 2.73 mm^3^/m, respectively (see Figure 15(a)). The zirconium substrate wear rate was higher than the composite coatings, signifying that composite coatings significantly improved the wear resistance of the Zr substrate. HA/GO/Pt annealed at 300°C shows to have a better wear resistance compared to the other samples.

The optical microscopic images of worn surface of HA/GO/Pt treated from 200 to 600°C under reciprocating movement with an applied normal load of 4N are shown in Figure 15(b). As can be seen, the thin film electrodeposited zirconium shows better resistance against a reciprocating load (wear) when the annealing temperature increases from 200 to 300°C. This may be due to an increase in adhesion strength between the thin film and the zirconium substrate and diffusion and penetration of the coated layer into the substrate. Moreover, hydroxyapatite in the composite coating plays as a lubricant with proper resistance to wear on the surface of the coated zirconium. Phosphate ions form hydrated ions in the thin film layer in which they turn into a large layer of hydration that results in nano/micro-ball baring lubricants between the coated substrate and the normal loads to increase the composite thin film antifriction property [35]. Nevertheless, research studies show that coatings obtain significant wear resistance with the inclusion of graphene oxide due to its high surface area and uniform dispersion which lets the energy be released from its uniform sites results in high fracture toughness of the composite thin layer of coating [36]. Also, crack energy dissipation, crack bridging, and propagation of crack will be mitigated because the crack can be deflected in a composite containing graphene (This has been investigated by Kvetkova et al.) [37]. Consequently, a continuous layer of graphene oxide formation has a significant effect on HA/GO/Pt thin film's resistance against wear.

The tribological properties of electrodeposited HA/rGO/Pd onto SST304 were studied in our previous research work [30]. The friction coefficient and wear resistance of heat-treated HA/rGO/Pd deposited stainless steel 304 at versatile temperatures were investigated. The coated substrate by HA/rGO/Pd treated at 600°C showed a noticeably lower average COF of 0.581 compared to other specimens. When the temperature increased from 200 to 400°C the friction coefficient increased and further temperature enhancement to 600°C resulted in a lower friction coefficient. Nevertheless, the coating showed better resistance against wear when the temperature enhanced from 200 to 600°C. This could be explained by diffusion of the coating into the substrate and enhance adhesion strength between the thin film and stainless steel 304 substrates. Compared to HA/rGO/Pd, treated HA/GO/Pt shows similar values for COFs, although the best COF occurred at 300°C in this study.

## 4. Conclusion

In this study, pure zirconium was selected as the substrate and HA, HA/GO, and HA/GO/Pt were the coatings deposited by electrodeposition technique. To evaluate the effect of annealing on properties of the composite coating, the HA/GO/Pt coated zirconium was heated at different annealing temperatures. A reciprocating ball on disc wear test machine was used to investigate the wear resistance of the composite coating after annealing. The coefficient of friction and wear rate of HA/GO/Pt coated substrate heated at 300°C were 0.54 and 2.02 mm^3^/m showing better results compared to the other samples heated at 200, 400, and 600°C. Furthermore, corrosion behavior and biocompatibility of HA/GO/Pt-, HA-, and HA/GO-coated zirconium were investigated. The biocompatibility result showed that the bare zirconium surface is capable of distribution of a very little number of cells. The biocompatibility experiments indicated that the coatings enhanced the cells attachments and proliferations atop the electrodeposited zirconium surface. HA/GO/Pt electrodeposited zirconium displayed insignificant cell attachment and proliferation compared to the other coatings. The electrochemical results showed that the HA/GO/Pt nanocomposite layer had better protection against corrosion compared to other coated specimens after soaking in the biomimetic synthetic medium. In addition, the electrochemical tests were conducted on the heat treated HA/GO/Pt electrodeposited zirconium and the results showed that the anodic current densities for samples annealed at 200, 300, 400, and 600°C are lower than the nonannealed HA/GO/Pt deposited zirconium. The best corrosion result was achieved at 400°C with I_corr_ and E_corr_ of 1.521 nA and 379.096 mV. This evidently proposes that utilizing HA/GO/Pt plus heat treatment reduces the risk of failure due to corrosion.

## Figures and Tables

**Table 1:** Current density, I_corr_, and corrosion potential, E_corr_, obtained from PDS measurements for an uncoated substrate and HA, HA/GO, and HA/GO/Pt coatings on zirconium after 7 days.

Time/day	Uncoated substrate	HA coating	HA/GO coating	HA/GO/Pt coating
I_corr_/nA				
7	279.602	72.1	50.044	27.227
E_corr_/mV				
7	-21.699	164.574	267.02	499.325

**Table 2:** Corrosion current density, Icorr, and corrosion potential, Ecorr, obtained from PDS measurements for corrosion behaviors of the thermal treated HA/GO/Pt coated zirconium.

Time/day	HT200	HT300	HT400	HT600
I_corr_/nA				
2	16.269	17.035	1.521	14.15
E_corr_/mV				
2	1.731	232.858	379.096	309.423

## Data Availability

The data including figures, graphs, and tables used to support the findings of this study are included in the article.
